# *Helicobacter pylori* Eradication on the Prevention of Metachronous Lesions after Endoscopic Resection of Gastric Neoplasm: A Meta-Analysis

**DOI:** 10.1371/journal.pone.0124725

**Published:** 2015-04-27

**Authors:** Da Hyun Jung, Jie-Hyun Kim, Hyun Soo Chung, Jun Chul Park, Sung Kwan Shin, Sang Kil Lee, Yong Chan Lee

**Affiliations:** 1 Department of Internal Medicine, Yonsei University College of Medicine, Seoul, Korea; 2 Department of Internal Medicine, Gangnam Severance Hospital, Yonsei University College of Medicine, Seoul, Korea; University of Texas MD Anderson Cancer Center, UNITED STATES

## Abstract

**Background:**

There is controversy about the effect of *Helicobacter pylori* (*H*. *pylori*) eradication on the prevention of metachronous gastric cancer after endoscopic resection (ER).

**Aims:**

The aim of this study was to systematically evaluate the effect of *H*. *pylori* eradication on the prevention of metachronous gastric lesions after ER of gastric neoplasms.

**Methods:**

We performed a systematic search of PubMed, EMBASE, the Cochrane Library, and MEDLINE that encompassed studies through April 2014. Our meta-analysis consisted of 10 studies, which included 5881 patients who underwent ER of gastric neoplasms.

**Results:**

When we compared the incidence of metachronous lesions between *H*. *pylori*-eradicated and non-eradicated groups, *H*. *pylori* eradication significantly lowered the risk of metachronous lesions after ER of gastric neoplasms (five studies, OR = 0.392, 95% CI 0.259 – 0.593, *P <* 0.001). When we compared *H*. *pylori*-eradicated and persistent groups, again, *H*. *pylori* eradication significantly lowered the incidence of metachronous lesions after ER of gastric neoplasms (six studies, OR = 0.468, 95% CI 0.326 – 0.673, *P <* 0.001). There was no obvious heterogeneity across the analyzed studies.

**Conclusions:**

This meta-analysis suggests a preventive role for *H*. *pylori* eradication for metachronous gastric lesions after ER of gastric neoplasms. Thus, *H*. *pylori* eradication should be considered if *H*. *pylori* infection is confirmed during ER.

## Introduction

The incidence of early gastric cancer (EGC) has been increasing as screening upper endoscopy has become widely available in Korea. The prognosis of EGC is quite favorable, with a 5 year survival rate > 95% [1]. Therefore, endoscopic resection (ER) has been a standard treatment for select cases of EGC in Korea. ER has many advantages, such as preservation of the stomach, quality of life, and reduced health costs. However, risk of metachronous gastric cancer in the remnant stomach after ER is higher than after gastrectomy [2]. The incidence of metachronous gastric cancer within 3–5 years after ER is 2.7–14.0% [3,4]. Therefore, scheduled endoscopic surveillance has been recommended to detect metachronous lesions after ER of EGC.

*Helicobacter pylori (H*. *pylori)* infection is closely related to progression to gastric dysplasia or cancer. In 1994, the International Agency for Research on Cancer (IARC), a subdivision of the World Health Organization (WHO), defined *H*. *pylori* as a group I carcinogen for gastric carcinoma [5]. However, the exact role of *H*. *pylori* infection in development of metachronous gastric lesions after ER has not been clearly elucidated. Fukase *et al*. reported that eradication of *H*. *pylori* after ER of EGC reduced the incidence of metachronous gastric cancer (odds ratio (OR) 0.353, 95% CI 0.161–0.775, *P* = 0.009), and recommended that prophylactic eradication should be pursued after ER [6]. However, Choi *et al*. showed that the incidence of metachronous cancer did not differ significantly between *H*. *pylori*-eradicated and control groups. This study enrolled 901 patients, who underwent ER for gastric dysplasia and cancer [7]. Thus, here lies the controversy about the effect of *H*. *pylori* eradication on prevention of metachronous gastric cancer after ER. Therefore, we aimed to systematically evaluate the effect of *H*. *pylori* eradication on prevention of metachronous gastric lesions after ER of gastric neoplasms.

## Methods

### Meta-analysis inclusion criteria

All relevant randomized controlled trials (RCTs) and retrospective cohort studies that compared the effects of *H*. *pylori* eradication on prevention of metachronous gastric lesions after ER of EGC were eligible for inclusion in our analysis.

### Identification of appropriate studies

PubMed (1966 to April 2014), Cochrane Library (1997 to April 2014), MEDLINE (1966 to April 2014), and EMBASE (1985 to April 2014) databases were queried during our computer-aided search. Database searches used the following terms: *Helicobacter pylori*, *H*. *pylori*, metachronous, second, recur, gastric dysplasia, neoplasm, and gastric cancer. We also searched references manually in order to not miss relevant articles. Two reviewers (DH Jung and J-H Kim) searched the databases independently. The primary outcome measure was the incidence of metachronous gastric lesions after *H*. *pylori* eradication.

### Study selection

Titles and abstracts were screened by two reviewers, and studies were chosen for meta-analysis if they were relevant. Language restrictions were not considered. If there was a disagreement, it was resolved by simultaneous review.

### Data extraction and quality assessment

Reviewers used standardized data extraction forms. Extracted data included baseline patient and tumor characteristics, status of *H*. *pylori* infection and eradication, duration of follow-up, and primary outcome measures reported by the authors. All obtained data were compared in order to minimize error.

### Measures of treatment effect

We compared the incidence of metachronous gastric neoplasms after ER of gastric neoplasms between *H*. *pylori*-eradicated and non-eradicated groups. We also compared the incidence of metachronous gastric neoplasms after ER of gastric neoplasms between *H*. *pylori*-eradicated and persistent groups. The results of each study were reported as a risk ratio (RR) between *H*. *pylori*-eradicated and non-eradicated or persistent groups, with a 95% confidence interval (CI).

### Assessment of heterogeneity

Statistical heterogeneity among trials was assessed with χ^2^ and *I* ^2^ tests. The *I* ^2^ test measures the percentage of variability between studies caused by heterogeneity but not chance. As values from the *I* ^2^ test increase, heterogeneity increases. Data were pooled according to the fixed-effects and random-effects models.

### Statistical analysis

The Begg’s funnel plot and Egger’s test were used to evaluate publication bias. *P* < 0.05 suggested a significant publication bias. Data was analyzed using CMA ver. 2.0 software (Comprehensive Meta-Analysis, Englewood, NJ, USA). Weights were assigned to individual studies based on the inverse of the variance. We used the PRISMA checklist (S1 PRISMA Checklist).

## Results

### Study inclusion

**Fig 1:**
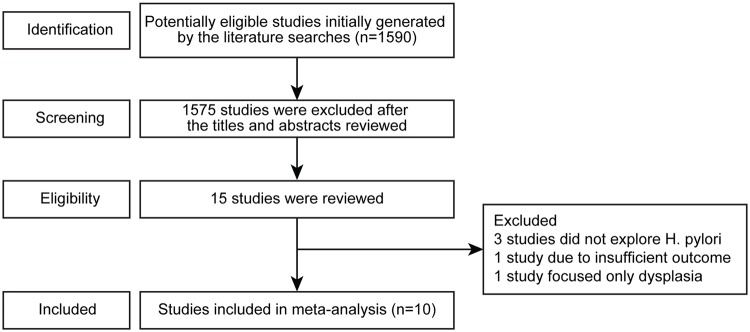
Flow chart for determining study inclusion .

Our literature search yielded a total of 10 studies associated with *H*. *pylori* eradication and metachronous gastric lesions that were included in the final analysis. Fig 1 shows the search process that resulted in the final selection of eligible studies. Of the 1590 studies identified through our search strategy, 1575 studies were excluded after review of titles and abstracts. The 15 articles that were potentially relevant were reviewed carefully. Of these, three studies were excluded because they did not explore *H*. *pylori* [8–10], and one study was excluded due to an insufficient description of metachronous gastric cancer [11]. The last study was excluded because it focused on patients with dysplasia [12].

### Heterogeneity

There was no heterogeneity for the primary outcome between *H*. *pylori*-eradicated and non-eradicated or persistent groups. There was no significant heterogeneity between *H*. *pylori*-eradicated and non-eradicated groups (χ^2^ = 3.11, *P* = 0.539, and *I* ^2^ = 0). Similarly, there was no significant heterogeneity between *H*. *pylori*-eradicated and persistent groups (χ^2^ = 2.05, *P* = 0.842, and *I* ^2^ = 0).

### Effect of *H*. *pylori* eradication on prevention of metachronous lesions after ER

**Fig 2:**
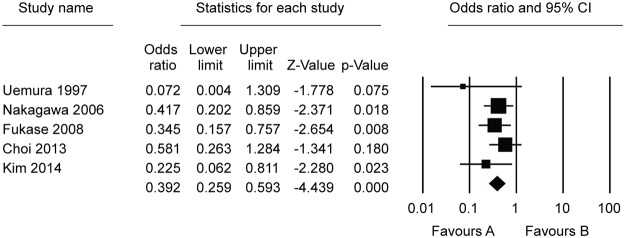
Forest plot showing comparisons for the effect of *Helicobacter pylori* eradication on metachronous gastric lesions after endoscopic resection between *Helicobacter pylori*-eradicated and non-eradicated groups.

**Fig 3:**
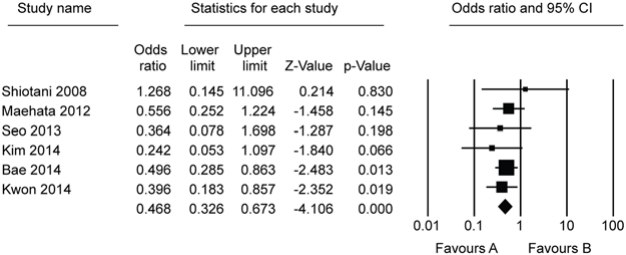
Forest plot showing comparisons for the effect of *Helicobacter pylori* eradication on metachronous gastric lesions after endoscopic resection between *Helicobacter pylori*-eradicated and persistent groups.

**Fig 4:**
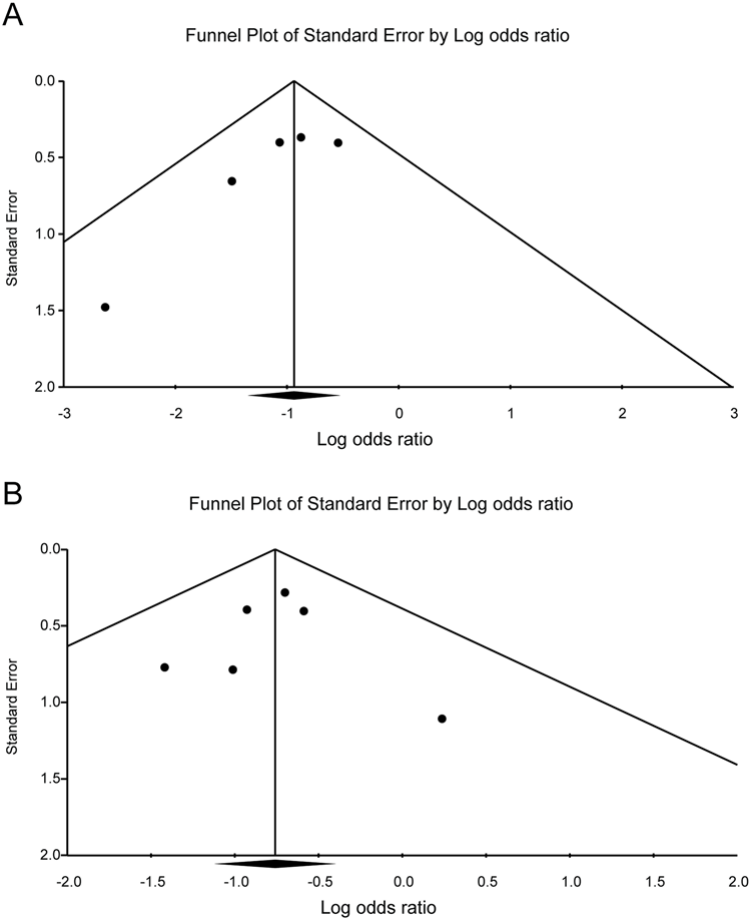
Publication bias plot for the effect of *Helicobacter pylori* eradication on metachronous gastric lesions after endoscopic resection. (A) comparison between *Helicobacter pylori*-eradicated and non-eradicated groups; (B) comparison between *Helicobacter pylori*-eradicated and persistent groups.

Ten studies, which included 5881 patients, compared the effect of *H*. *pylori* eradication on prevention of metachronous lesions after ER of gastric neoplasm (Table 1). Among these, five studies compared the incidence of metachronous lesions between *H*. *pylori*-eradicated and non-eradicated groups [6,7,13–15]. Six studies compared the incidence of metachronous lesions between *H*. *pylori*-eradicated and persistent groups [14,16–20]. One study compared the incidence of metachronous lesions between *H*. *pylori*-eradicated and non-eradicated or persistent groups [14]. On the whole, compared with the *H*. *pylori* non-eradicated group, results showed that *H*. *pylori* eradication was significantly helpful in preventing metachronous lesions after ER of gastric neoplasms (OR = 0.392, 95% CI 0.259–0.593, *P <* 0.001) (Fig 2). When we compared *H*. *pylori*-eradicated and persistent groups, *H*. *pylori* eradication significantly lower the incidence of metachronous lesions after ER of gastric neoplasms (OR = 0.468, 95% CI 0.326–0.673, *P <* 0.001) (Fig 3). According to the Begg’s and Egger’s tests, there was no apparent publication bias on the effect of *H*. *pylori* eradication for prevention of metachronous lesions after ER between *H*. *pylori*-eradicated and non-eradicated or persistent groups (Egger’s test, *P* = 0.090 or 0.926, funnel plot, Fig 4).

**Table 1:** Characteristics of studies that evaluated the effect of *Helicobacter*. *pylori* eradication on the prevention of metachronous gastric lesions after endoscopic resection of gastric neoplasm.

Study ID	Authors	Year	Ethnicity	Sample size (No receiving *H*. *pylori* eradication therapy)	Participant	Metachronous Recurrence	*H*. *pylori* Infection status (%)
1	Uemura et al.[15]	1997	Japanese	132 (67)	EGC	EGC	100
2	Nakagawa et al.[13]	2006	Japanese	2825 (2469)	EGC	EGC	100
3	Fukase et al.[6]	2008	Japanese	505 (250)	EGC	EGC	100
4	Shiotani et al.[16]	2008	Japanese	91 (0)	EGC	EGC	91.0
5	Maehata et al.[18]	2012	Japanese	268 (0)	EGC	EGC	100
6	Choi et al.[7]	2013	Korean	880 (441)	Gastric dysplasia or EGC	Gastric dysplasia or EGC	100
7	Seo et al.[17]	2013	Korean	74 (0)	EGC	EGC	100
8	Kim et al.[14]	2014	Korean	156 (88)	EGC	EGC	41.7
9	Bae et al.[19]	2014	Korean	667 (N/A)	EGC	EGC	66.2
10	Kwon et al.[20]	2014	Korean	283 (0)	EGC	Gastric dysplasia or EGC	69.0

*H*. *pylori*, *Helicobacter pylori*

EGC, early gastric cancer

### Sensitivity analysis

**Fig 5:**
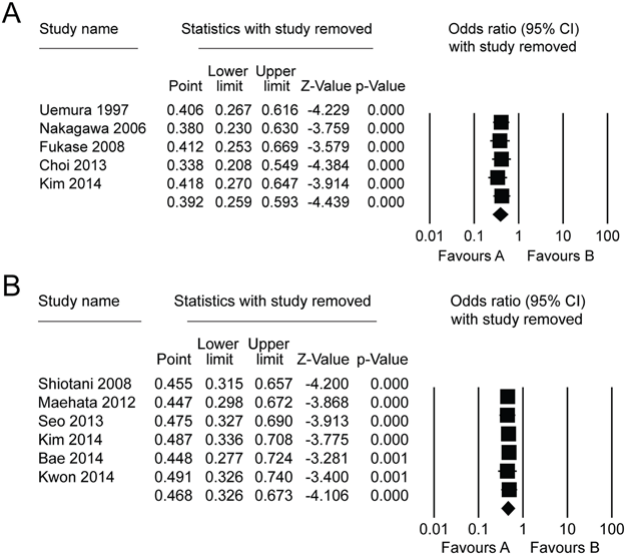
Sensitive analysis for the effect of *Helicobacter pylori* eradication on metachronous gastric lesions after endoscopic resection. (A) comparison between *Helicobacter pylori*-eradicated and non-eradicated groups; (B) comparison between *Helicobacter pylori*-eradicated and persistent groups.

A sensitivity analysis showed that the results of our meta-analysis could not be obviously influenced by removing any one study (Fig 5).

## Discussion

The effect of *H*. *pylori* eradication on the prevention of metachronous lesions after ER is still controversial. Thus, it may be useful to combine the results of similar published studies to arrive at a meaningful conclusion. As far as we know, this is the first meta-analysis to evaluate the association between *H*. *pylori* eradication and the incidence of metachronous lesions. Based on our findings, *H*. *pylori* eradication would be helpful for prevention of metachronous lesions after ER.

Nowadays, ER is widely used for local treatment of a gastric neoplasm. In Korea, the number of patients who have undergone ER for gastric neoplasm has increased annually because of the popularity of screening endoscopy [21]. The *H*. *pylori* infection rate in patients undergoing ER varies widely: 41.7–91.0% [14,16,19]. Our analysis suggests a preventive effect of *H*. *pylori* eradication since *H*. *pylori* eradication lowered the incidence of metachronous lesions after ER (OR = 0.392, 95% CI 0.259–0.593, *P* < 0.001). However, patients persistently infected after receiving *H*. *pylori* treatment were included in these groups. The study by Choi *et al*. showed the eradication rate of *H*. *pylori* after ER of gastric neoplasms [7]. Persistent *H*. *pylori* infection was found in 80 of 439 (18.2%) patients who received *H*. *pylori* treatment and in 373 of 441 (84.6%) patients who did not receive *H*. *pylori* treatment. We compared the effect of *H*. *pylori* treatment between *H*. *pylori*-eradicated and persistent groups. Successful *H*. *pylori* eradication was associated with a significant decrease in the incidence of metachronous lesions after ER (OR = 0.468, 95% CI 0.326–0.673, *P* < 0.001). This means that *H*. *pylori* eradication has a protective effect for the development of metachronous lesions. And, successful eradication of *H*. *pylori* is very important for the prevention of metachronous lesions after ER of a gastric neoplasm.

A large, prospective, randomized study in China reported that the incidence of gastric cancer was similar between patients receiving *H*. *pylori* eradication treatment and those receiving placebo. Subgroup analysis revealed that *H*. *pylori* eradication significantly inhibited development of gastric cancer in patients without a precancerous lesion [22]. However, several reports have shown that *H*. *pylori* eradication decreases the incidence of gastric cancer in high-risk patients as well [23,24]. Bae *et al*. reported that *H*. *pylori* eradication prevents development of metachronous lesions despite the presence of severe atrophy and intestinal metaplasia (IM) in the mucosal background [19].

Metachronous gastric cancer can develop after ER. Therefore, evaluating the risk factors associated with metachronous gastric cancer is important. Kwon *et al*. showed that old age and persistent *H*. *pylori* infection were independently significant risk factors for development of metachronous gastric cancer after ER of EGC [20]. Hanaoka *et al*. reported that extensive atrophic fundic gastritis diagnosed by autofluorescence imaging is a significant predictor for development of metachronous gastric cancer after *H*. *pylori* eradication [25]. According to Correa’s hypothesis, atrophic gastritis and IM caused by *H*. *pylori* infection are closely associated with the development of gastric cancer [26]. A meta-analysis of 12 studies inferred that *H*. *pylori* eradication significantly improved atrophic gastritis [27]. We cannot interrupt age-related atrophic changes in gastric mucosa. Thus, *H*. *pylori* eradication may be a very effective intervention strategy for promoting regression of metachronous lesions after ER.

Metachronous gastric cancers are found more frequently in patients following ER than in the gastrectomized stomach. It is caused naturally by the remnant stomach, which is preserved after ER. In addition, the surrounding non-tumorous mucosa may be at high risk of developing metachronous gastric lesions because it used to share the environment with gastric cancer [28]. Therefore, eradication of *H*. *pylori* should be recommended to promote regression of background mucosa in patients after ER of a gastric neoplasm.

Our study has some limitations. First, the ethnicity of study participants included in this meta-analysis was Korean and Japanese. The incidence of gastric cancer and *H*. *pylori* infection in Eastern Asia is higher than in other areas of the world. And, ER of gastric neoplasm is performed routinely in Korea and Japan [29–31]. Therefore, reports of an association between *H*. *pylori* eradication and metachronous recurrence after ER might be published mostly in Korea and Japan.

Secondly, our results did not segregate dysplasia and cancer. However, dysplasia was a pre-cancerous lesion as Correa’s hypothesis [26]. Therefore, to elucidate the effect of *H*. *pylori* eradication on prevention of metachronous lesions after ER is significant in patients with dysplasia or cancer.

In conclusion, the incidence of metachronous gastric cancer was higher in patients with persistent *H*. *pylori* infection than in those whose *H*. *pylori* infection was eradicated. And, eradication of *H*. *pylori* was helpful in decreasing the development of metachronous gastric cancer. Thus, eradication of *H*. *pylori* should be recommended if *H*. *pylori* infection is confirmed after ER.

## Supporting Information

S1 PRISMA Checklist (DOC)
